# Automatic configuration of the reference point method for fully automated multi‐objective treatment planning applied to oropharyngeal cancer

**DOI:** 10.1002/mp.14073

**Published:** 2020-03-05

**Authors:** Rens van Haveren, Ben J. M. Heijmen, Sebastiaan Breedveld

**Affiliations:** ^1^ Department of Radiation Oncology Erasmus MC University Medical Center Rotterdam 3015 GD Rotterdam The Netherlands

**Keywords:** automatic configuration, automated treatment planning, IMRT, oropharyngeal cancer, Pareto optimal, radiotherapy

## Abstract

**Purpose:**

In automated treatment planning, configuration of the underlying algorithm to generate high‐quality plans for all patients of a particular tumor type can be a major challenge. Often, a time‐consuming trial‐and‐error tuning procedure is required. The purpose of this paper is to automatically configure an automated treatment planning algorithm for oropharyngeal cancer patients.

**Methods:**

Recently, we proposed a new procedure to automatically configure the reference point method (RPM), a fast automatic multi‐objective treatment planning algorithm. With a well‐tuned configuration, the RPM generates a single Pareto optimal treatment plan with clinically favorable trade‐offs for each patient. The automatic configuration of the RPM requires a set of computed tomography (CT) scans with corresponding dose distributions for training. Previously, we demonstrated for prostate cancer planning with 12 objectives that training with only 9 patients resulted in high‐quality configurations. This paper further develops and explores the new automatic RPM configuration procedure for head and neck cancer planning with 22 objectives. Investigations were performed with planning CT scans of 105 previously treated unilateral or bilateral oropharyngeal cancer patients together with corresponding Pareto optimal treatment plans. These plans were generated with our clinically applied two‐phase *ε*‐constraint method (Erasmus‐iCycle) for automated multi‐objective treatment planning, ensuring consistent high quality and Pareto optimality of all plans. Clinically relevant, nonconvex criteria, such as dose‐volume parameters and NTCPs, were included to steer the RPM configuration.

**Results:**

Training sets with 20–50 patients were investigated. Even with 20 training plans, high‐quality configurations of the RPM were feasible. Automated plan generation with the automatically configured RPM resulted in Pareto optimal plans with overall similar or better quality than that of the Pareto optimal database plans.

**Conclusions:**

Automatic configuration of the RPM for automated treatment planning is feasible and drastically reduces the time and workload required when compared to manual tuning of an automated treatment planning algorithm.

## 1 Introduction

Generating high‐quality intensity‐modulated radiation therapy (IMRT) or volumetric modulated arc therapy (VMAT) treatment plans for oropharyngeal cancer patients is challenging. A high dose is to be delivered to the planning target volume (PTV), which is in close proximity to many critical surrounding organs‐at‐risk (OARs) such as salivary glands, oral cavity, swallowing muscles, larynx, esophagus, spinal cord, and brainstem.

Several automated treatment planning approaches have been proposed in the literature.^1^, ^2^ This paper focuses on automated multi‐objective fluence map optimization to generate a single Pareto optimal and clinically favorable treatment plan for each patient. Two algorithms for automated multi‐objective optimization of Pareto optimal plans have been developed in our center: (a) the two‐phase *ε*‐constraint (2p*ε*c) method^3^ which is part of the clinically applied Erasmus‐iCycle optimizer,^4^ and (b) the fast and fuzzy lexicographic reference point method^5^ (LRPM). Previous studies^6^, ^7^ have demonstrated that the quality of plans generated with the 2p*ε*c method is generally superior to that of manually generated plans. The main advantages of the LRPM over the 2p*ε*c method are faster plan generation with average relative speed‐up factors of 12 for prostate^5^ and 22 for head and neck cancer,^8^ and that trade‐offs between all planning objectives are balanced simultaneously (LRPM) instead of pairwise (2p*ε*c method), allowing for large gains for some objectives at the cost of minor degradations for other objectives.

However, algorithms for automated planning have to be configured separately for all tumor sites. Interactive (manual) tuning of the configuration is a time‐consuming and workload‐intensive procedure for both the 2p*ε*c method (“wish‐list” creation^4^, ^7^) and the LRPM.^5^, ^8^ Recently, we proposed a new automatic procedure^9^ to configure the reference point method^10^, ^11^, ^12^ (RPM), a special case of the LRPM.^5^ The procedure was successfully applied to prostate IMRT,^9^ and adaptive prostate and cervix IMPT.^13^, ^14^

This paper further develops and investigates the proposed automatic RPM configuration for a heterogeneous group of oropharyngeal cancer patients, with 22 objectives used in automatic plan generation. In previous work,^9^ creation and evaluation of RPM configurations was based on convex plan criteria. This paper investigates the use of clinically more relevant nonconvex criteria such as dose‐volume points or normal tissue complication probabilities (NTCPs). This allows for more flexible, intuitive, and clinically relevant automatic configurations. Dependency of the configuration quality on the (number of) selected training plans was included in the investigations.

## 2 Materials and Methods

### 2.1 Patient database

Planning CT scans of 105 previously treated unilateral and bilateral oropharyngeal cancer patients, together with a single corresponding Pareto optimal treatment plan per scan, were included in a database. All patients were treated with a simultaneously integrated boost technique similar to our clinical protocol.^15^ The high dose part of the PTV (PTV high) was prescribed 70 Gy, and the low dose part of the PTV (PTV low) was prescribed 54.25 Gy. A fixed coplanar equiangular 23 beam setup was used for each patient to mimic VMAT‐like dose distributions. The treatment was delivered in 35 fractions. In our clinical treatment planning workflow, the generated fluence map is automatically converted to a VMAT plan using Monaco (Elekta AB, Sweden). In this study however, plan comparisons are made with respect to the fluence maps so that the performance of both multi‐objective methods are objectively compared (no bias due to VMAT segmentation).

Each Pareto optimal plan in the database was generated with the 2p*ε*c method.^3^ The applied configuration (wish‐list) for plan generation with 2p*ε*c method is presented in Table 1. To achieve clinically acceptable coverage for both PTVs (V$95_{\%}\geq 98\%$), the logarithmic tumor control probability^16^ (LTCP) was used as the objective function. For the OARs, the focus was either on minimizing the mean dose (salivary glands, swallowing muscles, oral cavity, larynx, esophagus, and cochleas) or on minimizing the near maximum dose (spinal cord and brainstem) for which the generalized equivalent uniform dose^17^ (gEUD) with a high parameter value was used. Steering on the dose conformality was achieved by using maximum or near maximum doses to the PTV shells at 0, 5, 15, 30, 40, and 50 mm distance from the PTV. The entrance dose was controlled using the maximum dose to the external ring structure, which is the 20 mm ring inside the body contour. Hot spots were avoided by controlling the maximum dose in unspecified tissues.

**Table 1:** Wish‐list used for generating the database plans with the 2p*ε*c method. The down‐arrows (↓) indicate that the objectives are to be minimized. Prescribed dose was $D_{\mathrm{high}}\;=\;70$ Gy for the planning target volume (PTV) high, and $D_{\mathrm{low}}\;=\;54.25$ Gy for the PTV low

Volume	Type	Limit (Gy)	
Constraints			
PTV high	$D_{\max}$	74.9	(=107% of $D_{\mathrm{high}}$)
PTV high	$D_{\mathrm{mean}}$	70.7	(=101% of $D_{\mathrm{high}}$)
Spinal cord	$D_{\max}$	42	(=60% of $D_{\mathrm{high}}$)
Brainstem	$D_{\max}$	49	(=70% of $D_{\mathrm{high}}$)
PTV shell 0 mm	$D_{\max}$	70	(=100% of $D_{\mathrm{high}}$)
PTV shell 30 mm	$D_{\max}$	35	(=50% of $D_{\mathrm{high}}$)
Unspecified tissue	$D_{\max}$	74.9	(=107% of $D_{\mathrm{high}}$)

Priority	Volume	Type	Goal	Sufficient	Parameters
Objectives					
1	PTV high	↓LTCP	0.5	0.5	$D^{p}\;=\;D_{\mathrm{high}}$, *α* = 0.8
2	PTV low	↓LTCP	0.5	0.5	$D^{p}\;=\;D_{\mathrm{low}}$, *α* = 0.8
3	Parotid glands	$↓D_{\mathrm{mean}}$	20 Gy		
4	SMGs	$↓D_{\mathrm{mean}}$	35 Gy		
5	MCS/MCP	$↓D_{\mathrm{mean}}$	25 Gy		
6	MCM/MCI	$↓D_{\mathrm{mean}}$	25 Gy		
7	PTV shell 5 mm	$↓{\mathrm{gEUD}}_{10}$	10 Gy		
	PTV shell 15 mm	$↓{\mathrm{gEUD}}_{10}$	10 Gy		
8	Oral cavity/Larynx	$↓D_{\mathrm{mean}}$	35 Gy		
9	esophagus	$↓D_{\mathrm{mean}}$	40 Gy		
10	Spinal cord/brainstem	$↓{\mathrm{gEUD}}_{12}$	25 Gy		
11	PTV shell 40 mm	$↓{\mathrm{gEUD}}_{8}$	5 Gy		
	PTV shell 50 mm	$↓{\mathrm{gEUD}}_{8}$	5 Gy		
12	External ring 20 mm	$↓D_{\max}$	27.1 Gy		
13	Cochleas	$↓D_{\mathrm{mean}}$	35 Gy		

Abbreviations: gEUD_r_ = generalized equivalent uniform dose with applied parameter *r*; LTCP = logarithmic tumor control probability; MCI = musculus constrictor inferior; MCM = musculus constrictor medius; MCP = musculus constrictor cricopharyngeus; MCS = musculus constrictor superior; PTV = planning target volume; SMG = submandibular gland.

### 2.2 Automatic RPM configuration

**Figure 1:**
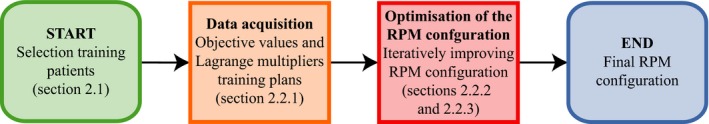
Schematic overview of the automatic reference point method configuration procedure. [Color figure can be viewed at http://wileyonlinelibrary.com]

The automatic RPM configuration procedure^9^ applied in this paper is summarized in Fig. 1. For initialization, a fraction of the patients in the database (Section 2.A) was randomly selected for training (the remaining test patients were used to validate the configuration). Then, relevant data were acquired from the training plans (Section 2.B.1) to create the final RPM configuration. (Sections 2.B.2 and 2.B.3).

#### 2.2.1 Data acquisition from training patients

The constraints and objectives used for plan generation with the 2p*ε*c method (Table 1) were also the basis for plan generation with the RPM. For an RPM configuration, two 22‐dimensional (or less if some OARs were not delineated) vectors were acquired from each training plan.

The first vector contained the values achieved for up to 22 objectives used in the fluence map optimization with the 2p*ε*c method (Table 1).

The other vector contained, for each objective, a quantity related to the overall trade‐offs made. More specifically, these were the Lagrange multipliers (one for each objective) resulting from the fluence map optimization with the 2p*ε*c method. These Lagrange multipliers can be found as a byproduct of the optimization.^3^, ^9^

#### 2.2.2 Automatic configuration procedure

The RPM automatically generates a fluence map by solving the minimization problem

(1) $$\underset{x\in X}{\text{minimize}}\left\{\max_{i\in [n]}[w_{i}f_{i}\left(x\right)+c_{i}]+\sum_{i\in [n]}\rho_{i}(w_{i}f_{i}\left(x\right)+c_{i})\right\}.$$

Here, *x* is the fluence map, *X* a constrained set, $f_{1}\left(x\right),\ldots ,f_{n}\left(x\right)$ the objectives, and the $w_{1},c_{1},\rho_{1}\ldots ,w_{n},c_{n},\rho_{n}$ define an RPM configuration. The $w_{1},c_{1},\ldots ,w_{n},c_{n}$ prioritise the objectives, and $\rho_{1},\ldots ,\rho_{n}$ quantify desired trade‐offs between objectives. In the automatic procedure, each RPM configuration is iteratively generated. In the first iteration, the data acquired from the training database (Section 2.B.1) was used to generate an initial RPM configuration (technical details^9^). With this configuration, a single Pareto optimal RPM plan can then be automatically generated for each training patient. Based on the differences observed between training and RPM‐generated plans for target coverage and other plan parameters, the RPM configuration was then either accepted or not (see Section 2.B.3). If an RPM configuration was not accepted, the configuration was updated for the next iteration and the process was repeated. Updating the configuration is achieved by updating the trade‐off parameters $\rho_{1},\ldots ,\rho_{n}$. The general rule is that $\rho_{i}$ is increased if its corresponding plan parameter scored worse than desired, but is decreased if the corresponding plan parameter scored better for the population than desired (details of the heuristic^9^). If an RPM configuration was acceptable or if the RPM configuration is still not acceptable after 40 iterations (heuristic), the iterative process terminated and returned the final RPM configuration.

#### 2.2.3 User‐defined preferences for automatic RPM configuration

Each automatic RPM configuration is steered by a set of user‐defined preferences. There are two types of preferences: (a) preferences regarding a minimum/maximum allowed value for a plan parameter in the RPM‐generated plans; (b) preferences regarding differences for a plan parameter between the training and RPM‐generated plans. An example for the first type is seen in the first row of Table 2, which indicates that the minimum allowed value for V$95_{\%}$ of the PTV low and PTV high is 98% in all RPM‐generated plans. An example of the second type is seen in the second row of Table 2, which indicates that the median value of all differences (database − RPM) in the parotid gland NTCP (for both left and right) is at least 0 (in %‐point). Instead of the median value, other percentile values can be used as well. Multiple measures can be defined per plan parameter. If all measures are above the desired lower bounds, the RPM configuration is accepted.

**Table 2:** User preferences to create and evaluate an reference point method (RPM) configuration

Plan parameter	Type	Lower bound	Planning objective
PTV low/PTV high V$95_{\%}$	Minimum	98	PTVs LTCP
Parotids glands NTCP	Median	0	Parotid glands $D_{\mathrm{mean}}$
	5th percentile	−2.5	
SMGs/oral cavity NTCP	Median	0	SMGs/oral cavity $D_{\mathrm{mean}}$
	5th percentile	−4	
MCS $D_{\mathrm{mean}}$	Median	0	
	5th percentile	−2	
MCP $D_{\mathrm{mean}}$	Median	0	
	5th percentile	−2.5	
MCM/MCI $D_{\mathrm{mean}}$	Median	0	
	5th percentile	−3	
Larynx/esophagus $D_{\mathrm{mean}}$	Median	0	
	5th percentile	−3	
Spinal cord/brainstem gEUD_12_	Median	−1	
	5th percentile	−3	
Cochleas $D_{\mathrm{mean}}$	1st quartile	−5	
PTV shell 5 mm gEUD_8_	Median	−0.5	
	5th percentile	−3	
PTV shell 15 mm gEUD_8_	Median	−0.75	
	5th percentile	−3.25	
PTV shell 40 mm gEUD_8_	Median	−1.25	
	5th percentile	−3.75	
PTV shell 50 mm gEUD_8_	Median	−1.5	
	5th percentile	−4	
External ring 20 mm $D_{\max}$	Median	−1.5	
	5th percentile	−5	

1st quartile  = 25th percentile; gEUD_*r*_ = generalized equivalent uniform dose with applied parameter *r*; LTCP = logarithmic tumor control probability; MCI = musculus constrictor inferior; MCM = musculus constrictor medius; MCP = musculus constrictor cricopharyngeus; MCS = musculus constrictor superior; Median = 50th percentile; PTV = planning target volume; SMG = submandibular gland.

For the automatic RPM configuration applied to prostate cancer,^9^ only the convex constraints and objectives as applied in the wish list were used for defining the user preferences. A drawback of this approach is that clinically relevant plan quality criteria may involve nonconvex functions such as dose‐volume points or models for predicting NTCPs. Therefore, we extended the previous methodology by allowing general nonconvex functions to be applied in the user preferences. The user preferences in Table 2 were applied for creation and evaluation of all RPM configurations. The applied nonconvex functions were linked to convex surrogates, which were used in the plan optimizations (compare with Table 1). The first row in Table 2 specifies that for both the PTV high and PTV low, the V$95_{\%}$ should be at least 98% in all RPM‐generated plans. The following Lyman NTCP model^18^ was applied for predicting xerostomia,

(2) $$\mathrm{NTCP}\left(D_{\mathrm{mean}}\right)={\left(2\pi \right)}^{-1/2}\int_{-\infty }^{(D_{\mathrm{mean}}-40)/16}\exp\left(-t^{2}/2\right)dt,$$

with $D_{\mathrm{mean}}$ being the mean dose in a salivary gland or the oral cavity. The second row in Table 2 specifies that the NTCP values in at least 50% of the RPM‐generated plans should be lower than those in the training plans, and that the NTCP values in at most 5% of the RPM‐generated plans can be 2.5%‐points higher than those in the training plans.

The aim of the user preferences in Table 2 was to define an RPM configuration resulting in plans with: (a) sufficient target coverage for all patients (V$95_{\%}\geq 98\%$); (b) overall reduced NTCP values in salivary glands and oral cavity and reduced mean doses in the swallowing muscles. If needed to accomplish (a) and (b), moderate deteriorations were allowed for the spinal cord, brainstem, cochleas, and conformality measures (PTV shells and external ring). Both the median and 5th percentile were often used to both control the overall differences and to mostly avoid large unfavorable outliers for the RPM.

### 2.3 Variations in training sets

**Figure 2:**
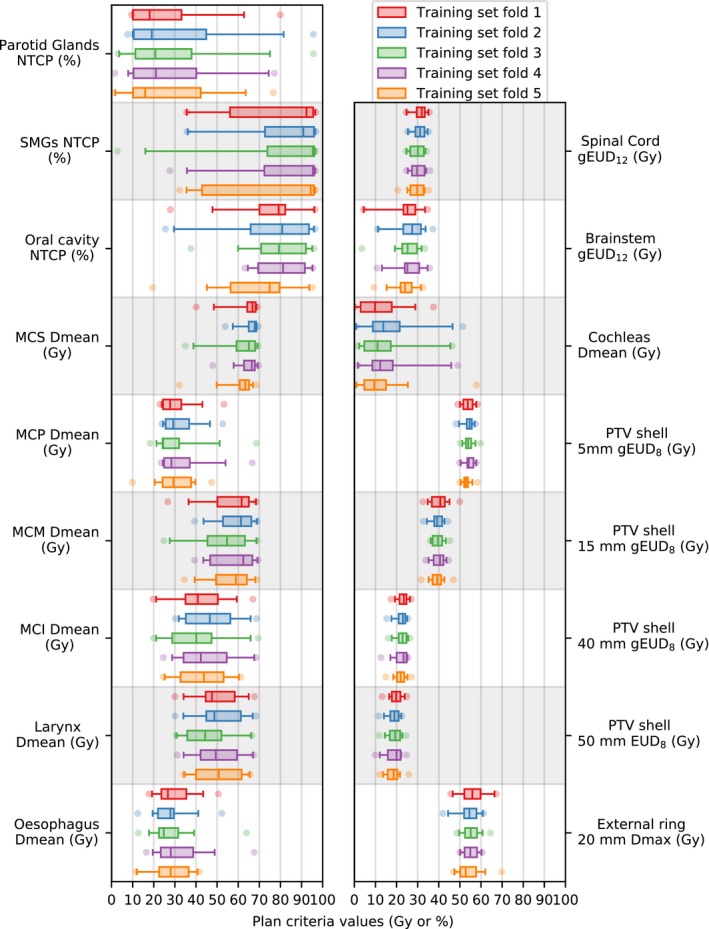
Boxplots of the plan parameter values (Table 2) for the database plans corresponding to the five different training folds, each with 20 training patients. Vertical thick lines within the boxes are medians, boxes are between the first and third quartile, whiskers are between the 2.5th and 97.5th percentile, circles are outliers. [Color figure can be viewed at http://wileyonlinelibrary.com]

RPM configurations were established for various training sets: a variation of *k*‐fold cross‐validation was applied to training sets with 20 (*k* = 5) plans. Training sets with 35 (*k* = 3) and 50 (*k* = 2) plans were also tested. Selection of patients for the training folds was always random, with each patient only present in one fold. The quality of an RPM configuration was determined by comparing the RPM‐generated plans with the database plans for the test patients (patients not used for training) regarding the plan parameters defined in Table 2. To visualize the heterogeneity of the training folds with 20 plans, the plan parameters for the corresponding database plans are shown in Fig. 2.

Paired two‐sided Wilcoxon signed rank tests were applied to assess whether or not the differences in plan parameter values between database and RPM‐generated plans for the test patients were statistically significant (*P* < 0.05).

## 3 Results

### 3.1 Target coverage

All database and RPM‐generated plans had clinically acceptable target coverage, that is, the V$95_{\%}$ was at least 98% for both the PTV high and the PTV low.

Differences in target coverage between the database and corresponding RPM‐generated plans up to 1%‐point were observed. To focus on analyzing differences in other plan parameters, all dose distributions were first scaled such that the V$95_{\%}$ for either the PTV low or PTV high was 98%.

### 3.2 OAR sparing and conformality

**Figure 3:**
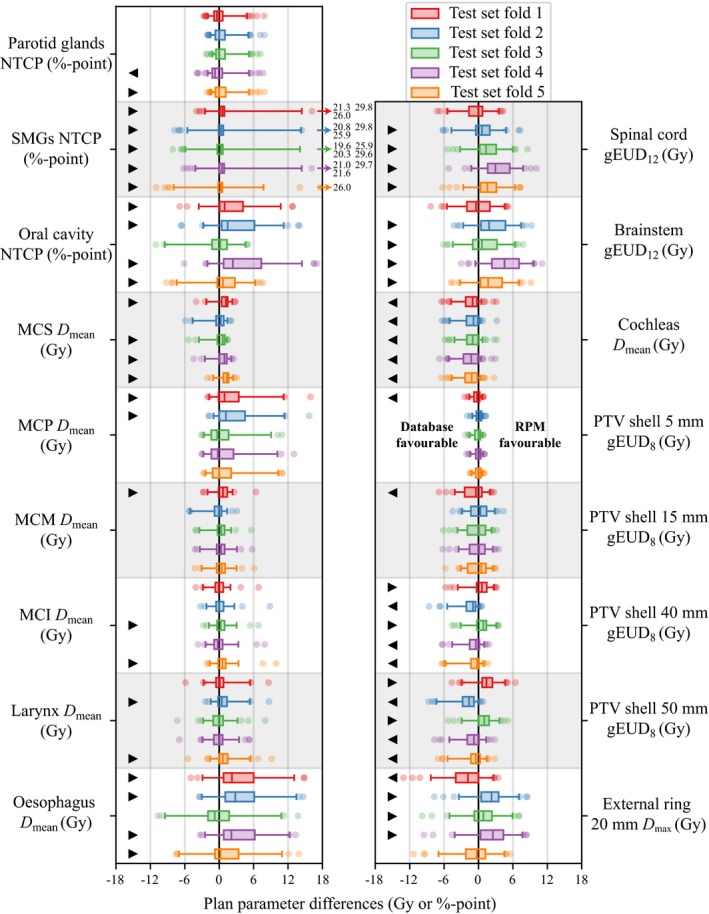
Boxplots of the differences in plan parameter values (Table 2) between database plans and reference point method (RPM)‐generated plans for the five test folds corresponding to the five different RPM configurations with 20 training patients. Positive values are favorable for the RPM. Vertical thick lines within the boxes are medians, boxes are between the first and third quartile, whiskers are between the 2.5th and 97.5th percentile, circles are outliers, arrows indicate large outliers. Statistically significant differences (*P* < 0.05) in favor of database plans (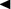) or RPM plans (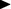). [Color figure can be viewed at http://wileyonlinelibrary.com]

For the five RPM configurations based on different sets of 20 training patients, the differences observed in plan parameters between database and RPM‐generated plans for the test patients are presented in Fig. 3.

For most plan parameters, the distribution of differences and the corresponding median difference for the five test folds were similar. The submandibular glands (SMGs), oral cavity, esophagus, spinal cord, and brainstem showed overall better sparing for the RPM‐generated plans at the cost of some deterioration in conformality measures. Differences observed in plan parameters between RPM‐generated plans and database plans are in line with Table 2, where preference is given to improve RPM‐generated plans regarding organ sparing by allowing some deterioration in conformality.

**Figure 4:**
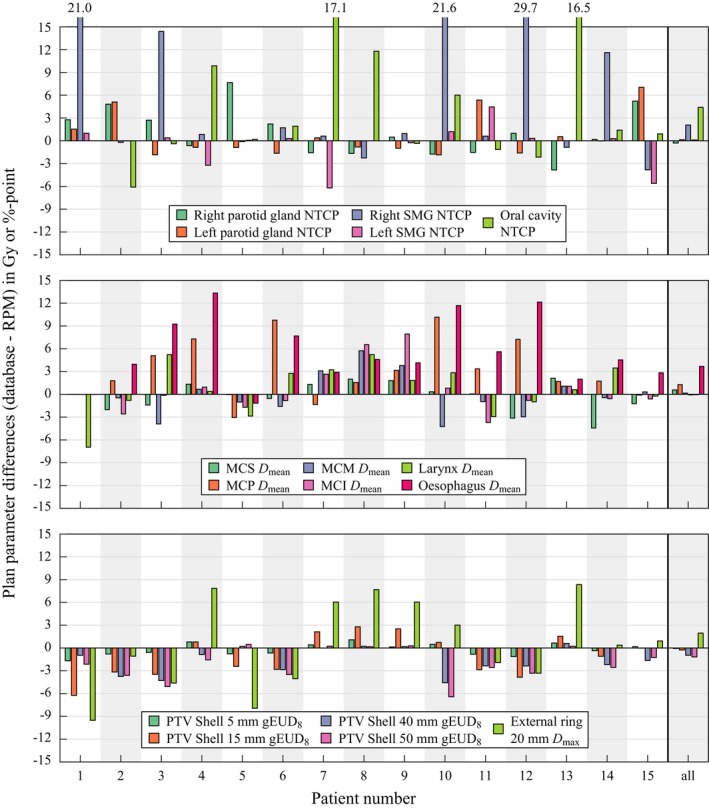
Differences in most important plan parameter values between database plans and reference point method (RPM)‐generated plans for the 15 most extreme outliers in test fold 4 (training with 20 patients), both favorable and unfavorable for the RPM. Positive values are favorable for the RPM. The last column shows the average results for all test patients. [Color figure can be viewed at http://wileyonlinelibrary.com]

Figure 3 also shows outliers for some plan parameters, often in favor of the RPM. Since the performance of the RPM configuration (obtained with training set fold 4, see Fig. 2) on test fold 4 (see Fig. 3) is according to the user preferences (Table 2), particularly for the oral cavity NTCP, this fold is analyzed more in depth. For test fold 4, the differences in the most important plan parameters are shown in Fig. 4 for 15 plans with the most extreme outliers (both favorable and unfavorable for the RPM). As a reference, the last column in Fig. 4 shows the mean differences for *all* test fold 4 patients, clearly showing an overall gain for the RPM.

**Figure 5:**
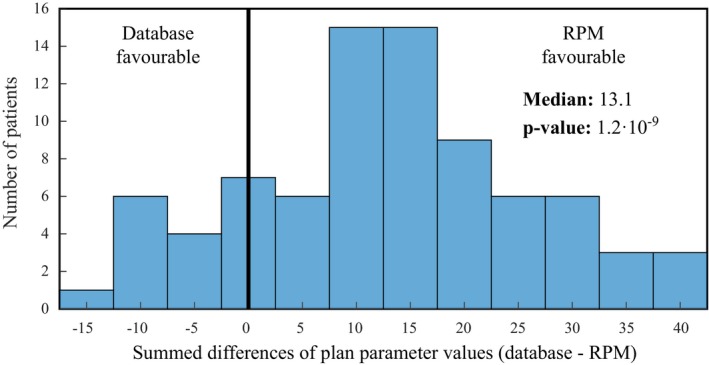
Histogram of the summed differences of plan parameter values (database — reference point method) for all 85 patients in test fold 4 (training with 20 patients). The median gain of 13.1 indicates an advantage for the RPM (*P* < 0.001). [Color figure can be viewed at http://wileyonlinelibrary.com]

In another approach for comparing RPM‐generated plans with database plans, differences in all plan parameter values were summed for each patient in test fold 4. A histogram of the summed differences is presented in Fig. 5. The median of the summed differences was 13.1, indicating an advantage for the RPM (*P* < 0.001). This advantage was seen in 70 out of 85 patients in test fold 4.

In the supplementary material, results are presented for training with 35 and 50 patients. In general, it was found that increasing the number of training patients resulted in (a) slightly more consistent results for the test patients among the different folds with the same number of training patients, and (b) reduced severity of the outliers unfavorable for the RPM.

### 3.3 Computation times

All computations were performed on a dual Intel Xeon E5‐2690 Linux server using an in‐house developed solver tuned for radiotherapy treatment planning.^19^ On average, 5.6 min of computation time was required to generate a single RPM plan. Total computation times to automatically generate an RPM configuration ranged between 22.3 and 61.9 h without any user interaction.

## 4 Discussion

The purpose of this study was to further develop and explore a recently introduced automatic configuration procedure for the RPM,^9^ an algorithm for fast automated multi‐objective treatment planning. The automatic configuration procedure requires a training set (delineated CT scans with corresponding treatment plans) as input. This study tested the automatic configuration for a heterogeneous group of unilateral and bilateral oropharyngeal cancer patients with planning based on 22 objectives, and demonstrated that high‐quality configurations were obtained with only 20 training patients.

In previous work,^8^ the LRPM was used to automatically generate clinically favorable treatment plans for fifteen head and neck cancer patients. In that paper, part of the LRPM configuration (trade‐off configuration) was established manually. This study improves on that work in several ways. First, we have shown that clinically favorable treatment plans for head and neck cancer patients can also be generated with the RPM (linear reference path) instead of the more complex LRPM (piecewise linear reference path). Secondly, it was shown that a single RPM configuration can generate clinically favorable plans for a larger patient database (105 patients instead of 15). Thirdly, in this work, the RPM configuration was automatically generated, removing the need for extensive manual tuning. Finally, a more heterogeneous patient database was included in this study, demonstrating flexibility of the RPM for automated treatment planning.

Whereas the user preferences for creating and evaluating RPM configurations in previous work^9^ were based exclusively on the convex planning objectives used in fluence map optimization, this paper describes how nonconvex criteria, such as dose‐volume parameters or NTCPs (e.g., see Table 2), can be included by coupling them to correlated convex objectives. This made the automatic configuration more intuitive and clinically relevant, while the fluence map optimization problem remained convex guaranteeing optimality of the plan generated.

Automatic RPM configurations were based on user preferences regarding population‐based differences between database and RPM‐generated plans (e.g., Table 2). In practice, the lower bounds defined for the statistical population‐based user preferences can be derived iteratively. For example, the first step can be to only define a median for each criterion, then perform a full configuration run, and then add or adjust the measures and lower bounds for criteria that showed undesired trade‐offs. In this way, the user iteratively gets a better understanding about which of the plan parameters are difficult to improve, and which are less difficult to improve. This procedure can then be repeated until a configuration is obtained that results in desirable trade‐offs between all criteria. Tuning the entries in Table 2 is easier for the user than tuning the RPM parameters directly, since the user is familiar with interpreting the plan parameters but not with the RPM parameters. Even with expert knowledge of the RPM, automatic configuration has shown to be superior^5^, ^9^ for prostate planning. Note that for any configuration, Pareto optimality of all RPM‐generated plans is guaranteed.^11^

Compared to the automatic RPM configuration for automatic prostate planning,^9^ we observed more variation in differences between database and RPM‐generated plans among the training folds (Fig. 3 and Figs. S1 and S2) For example, for the different training sets of 20 patients (Fig. 3), slightly different trade‐offs were observed among the different test folds: folds 1 and 2 showed better sparing of SMGs and oral cavity than folds 3 and 5 at the cost of degradations in the conformality measures. For training based on a larger training set of 50 patients (Fig. S2), the median differences were more consistent among the different test folds. However, differences in outliers were still present: fold 1 showed better sparing of SMGs and oral cavity than fold 2. This is likely due to the heterogeneous patient database (Section 2.A). As can be seen in Fig. S2, the distribution of differences in plan parameter values for the test patients in fold 2 were slightly worse than desired (Table 2) for the SMGs and oral cavity NTCP values. The recommendation for a heterogeneous group of patients is to generate various configurations, one for each different training fold, also with variation in training set sizes, in order to investigate variation in configuration quality related to the patient heterogeneity. Each of these configurations could include an iterative fine‐tuning of the user preferences (see above and Table 2). A single (large) test fold could ideally be the basis for all configurations (requiring many patients). Ideally, there is also a large evaluation fold with patients not used for |commentAUTHOR: Please check the sentence \x93patients not used for training \x85\x94 for sense and clarity.training nor testing for final configuration selection and quality assessment.

Overall, the RPM‐generated plans showed a better OAR sparing at the cost of some decreased conformality. In Fig. 5, differences in OAR criteria values were added for each test plan and displayed in a histogram. The median improvement of 13.1 units is in favor of the RPM (*P* < 0.001). Technically, the maximum gain for this measure can be achieved by generating plans using the weighted sum method with equal weights.^20^ However, the RPM also ensures that the differences in criteria values corresponding to OARs with high clinical priorities are within an acceptable range for each patient, which can be observed in Fig. 3.

A similar approach to automatic configuration of the RPM is knowledge‐based planning^21^ (KBP). Both approaches rely on a set of training plans from previously treated patients. The main difference is that the training plans lead to explicit specification of the RPM parameters in the automatic configuration approach, while they are applied to create a model in the KBP approach. This model is trained, using machine learning techniques such as deep‐learning,^22^ support vector regression^23^ or generative adversarial networks,^24^ to predict the DVHs or spatial dose distribution prediction. The predicted DVHs or dose distribution are then the basis for plan optimization.^25^, ^26^, ^27^ Both the automatic configuration and the KBP approach report promising results.

The RPM automatically generates a Pareto optimal fluence map plan and can thus not be directly delivered as the treatment device parameters are still unspecified. A recently developed automated segmentation algorithm^28^ shows segmented plans are dosimetrically similar to the fluence map plans. The plan comparisons presented in this paper should therefore be an accurate representation of the plan comparisons after segmentation.

In this paper, the objectives and constraints in Table 1 were used as a starting point for all automatic plan generations. A next step can be to eliminate the requirement to explicitly specify these objectives and constraints, which could possibly be achieved with inverse multi‐objective optimization techniques.^29^ This is a topic for further research.

## 5 Conclusions

A fully automated procedure for flexible and intuitive configuration of the reference point method (RPM), an algorithm for fast automated multi‐objective plan generation, was tested for a heterogeneous group of oropharyngeal cancer patients. For each patient, the automatic RPM configuration allowed for fast automatic generation of a Pareto optimal plan with clinically favorable trade‐offs, even for configurations based on only 20 training patients. As requested, the configurations generally resulted in lower OAR doses than those in the database plans at the cost of slightly reduced conformality. The RPM also resulted in favorable outliers for doses in highly prioritized OARs. Automatic RPM configuration has great potential in replacing traditional time‐consuming and labor‐intensive treatment planning workflows relying on manual configuration.

## Conflict of Interest

The Erasmus MC Cancer Institute has research collaborations with Elekta AB, Stockholm, Sweden and Accuray Inc., Sunnyvale, USA. These companies were not involved in the work in this paper. The authors have no conflict to disclose.

## Supporting information


**Figure. S1.** Boxplots of the differences in plan parameter values (Table 2) between database plans and RPM generated plans for the three test folds corresponding to the three different RPM configurations with 35 training patients. Positive values are favourable for the RPM. Vertical thick lines within the boxes are medians, boxes are between the first and third quartile, whiskers are between the 2.5th and 97.5th percentile, circles are outliers, arrows indicate large outliers. Statistically significant differences (*P* < 0.05) in favour of database plans (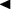) or RPM plans (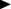).

**Figure. S2.** Boxplots of the differences in plan parameter values (Table 2) between database plans and RPM generated plans for the two test folds corresponding to the two different RPM configurations with 50 training patients. Positive values are favourable for the RPM. Vertical thick lines within the boxes are medians, boxes are between the first and third quartile, whiskers are between the 2.5th and 97.5th percentile, circles are outliers, arrows indicate large outliers. Statistically significant differences (*P* < 0.05) in favour of database plans (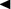) or RPM plans (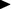).
